# Susceptibility to geometrical visual illusions in Parkinson’s disorder

**DOI:** 10.3389/fpsyg.2023.1289160

**Published:** 2024-01-08

**Authors:** Radoslaw Wincza, Calum Hartley, Megan Readman, Sally Linkenauger, Trevor Crawford

**Affiliations:** ^1^Department of Psychology, Lancaster University, Lancaster, United Kingdom; ^2^University of Liverpool, Liverpool, United Kingdom

**Keywords:** Parkinson’s disorder, visual illusions, Ebbinghaus illusion, Ponzo illusion, Müller-Lyer illusion, depth perception

## Abstract

Parkinson’s disorder (PD) is a common neurodegenerative disorder affecting approximately 1–3% of the population aged 60 years and older. In addition to motor difficulties, PD is also marked by visual disturbances, including depth perception, abnormalities in basal ganglia functioning, and dopamine deficiency. Reduced ability to perceive depth has been linked to an increased risk of falling in this population. The purpose of this paper was to determine whether disturbances in PD patients’ visual processing manifest through atypical performance on visual illusion (VI) tasks. This insight will advance understanding of high-level perception in PD, as well as indicate the role of dopamine deficiency and basal ganglia pathophysiology in VIs susceptibility. Groups of 28 PD patients (*Mage* = 63.46, *SD* = 7.55) and 28 neurotypical controls (*Mage* = 63.18, *SD* = 9.39) matched on age, general cognitive abilities (memory, numeracy, attention, language), and mood responded to Ebbinghaus, Ponzo, and Müller-Lyer illusions in a computer-based task. Our results revealed no reliable differences in VI susceptibility between PD and neurotypical groups. In the early- to mid-stage of PD, abnormalities of the basal ganglia and dopamine deficiency are unlikely to be involved in top-down processing or depth perception, which are both thought to be related to VI susceptibility. Furthermore, depth-related issues experienced by PD patients (e.g., increased risk for falling) may not be subserved by the same cognitive mechanisms as VIs. Further research is needed to investigate if more explicit presentations of illusory depth are affected in PD, which might help to understand the depth processing deficits in PD.

## Introduction

Visual illusions (VIs) occur when the configuration of a stimulus causes the viewer to incorrectly perceive relationships between its parts (Notredame et al., 2014). VIs have been widely used as a tool to investigate how visual perception develops (e.g., Doherty et al., 2010) and the impact of neuropsychological disorders such as schizophrenia (for a review see King et al., 2016; Costa et al., 2023) and autism (for a review see Gori et al., 2016). Although impairment of visual perception (e.g., hallucinations) is now well established in Parkinson’s disorder (PD) (Sauerbier and Ray Chaudhuri, 2013; Weil et al., 2016; Nieto-Escamez et al., 2023), research has yet to investigate how PD affects susceptibility to VIs. Furthermore, depth perception—which is linked to VI susceptibility (e.g., Gregory, 1963; Doherty et al., 2010; Gregory, 2015) and increased risk of falling (Cummings et al., 1995)—is shown to be affected in PD (Maschke et al., 2006). Therefore, studying VI susceptibility in this population may indicate how neuropsychological characteristics of PD (e.g., dopamine deficits and the pathophysiology of the basal ganglia) impact depth perception and top-down visual processing.

PD is a common neurodegenerative disorder affecting approximately 1–3% of the population aged 60 years and older (Pringsheim et al., 2014; Ball et al., 2019). It is characterized by motor deficits including tremors, rigidity, bradykinesia (slowed movement execution and initiation), and postural instability (Berardelli et al., 1983; Guttman et al., 2003). Although PD was traditionally considered to be a paradigmatic motor disorder, non-motor disruptions (including visual distortions) are experienced by the majority of PD patients (Chaudhuri et al., 2011). Visual distortions in PD include decreased contrast sensitivity (Uc et al., 2005; Sauerbier and Ray Chaudhuri, 2013; van der Lijn et al., 2022), decreased color discrimination (Pieri et al., 2000), deficits in motion and spatial perception (Uc et al., 2005), visual acuity deficits (Uc et al., 2005), and visual hallucinations (Barnes, 2001; Weil et al., 2016).

It is widely regarded that visual disturbances in PD are caused by a reduction of dopamine (Bodis-Wollner, 1990). Dopamine, a key neurotransmitter in the mammalian brain (Bibb, 2005), is believed to play a crucial role in visual perception (Harris et al., 2003). For example, Andreou et al. (2015) showed that dopamine influences neurotypical adults’ sensitivity to detecting an object in snowy (noisy) black-and-white pictures. Dopamine has also been shown to influence visual perception in PD. Multiple studies have found that retinal dopamine levels and dopaminergic innervation surrounding the fovea are reduced in PD (Harnois and Di Paolo, 1990; Sauerbier and Ray Chaudhuri, 2013; Nieto-Escamez et al., 2023), resulting in visual perception deficits such as poorer light adaptation and decreased contrast sensitivity (e.g., Pieri et al., 2000; Armstrong, 2015). Other visual deficits that are linked to dopamine deficiency include greater thresholds for motion detection (e.g., Trick et al., 1994), color discrimination (e.g., Büttner et al., 1994), as well as visuospatial deficits (e.g., Gibson et al., 1987; for an overview of dopamine-related deficits in PD, see Brandies and Yehuda, 2008).

Another hallmark of PD is the pathophysiology of the basal ganglia (Obeso et al., 2000). The basal ganglia are believed to control motor and cognitive functioning (Macpherson and Hikida, 2019); however, recent research has implicated their role in visual perception (Maschke et al., 2006; Nieto-Escamez et al., 2023). Maschke et al. (2006) showed that PD patients and patients with spinocerebellar ataxia (a movement disorder) made greater errors when estimating the slant of an illusory display (Ames Trapezoidal Window). The difficulties evidenced by PD patients were attributed to differences in the basal ganglia’s functioning. Furthermore, dopamine losses across key components of the basal ganglia (e.g., subthalamic nucleus, substantia nigra, and globus pallidus) are observed in PD (Benazzouz et al., 2014). Dopamine deficiency in the basal ganglia is of particular interest, as the link between these two is thought to be related to the processing of visual information. Sil’kis (2007) proposed a mechanism in which the basal ganglia modulates the efficiency of synaptic transmission in an interconnected parallel circuit that involves the limbic cortex, basal ganglia, thalamus, and cortex. This process is contingent on dopamine-dependent processes. It is, therefore, plausible to suspect that changes to this circuit in PD, could result in abnormal VIs susceptibility.

Given the well-documented abnormalities in depth perception in PD (Maschke et al., 2006; Ou et al., 2018), which could be linked to dopamine deficiency and the role of the basal ganglia (e.g., Maschke et al., 2006), it may be that susceptibility to depth-related VIs (e.g., the Ponzo illusion) is atypical in this population. Studying VIs in PD will enable us to comprehend the potential relationship between dopamine losses and basal ganglia pathophysiology with susceptibility to VIs. Consequently, VIs could offer a promising approach to address perceptual depth deficits in PD.

Although abnormalities in the basal ganglia and deficiency in dopamine levels could potentially influence sensitivity to depth-related VIs in PD, there are reasons to believe that sensitivity to *high-level* VIs may be preserved. The term “high-level VIs” is used to classify illusions that are thought to emerge at a later stage of visual processing (from approximately the V1 and beyond) compared to low-level illusions that are mediated at the retinal level and up to V1 (King et al., 2016). The Ebbinghaus, Ponzo, and Müller-Lyer are examples of high-level illusions, while the Brightness and Herman Grid illusions are examples of low-level illusions (King et al., 2016).

Goodale and Milner (1992) classic theory proposes that there are two visual streams in the brain. The ventral stream is responsible for perception for vision, while the dorsal stream is responsible for perception for action. VIs represent a unique method for investigating differences between these two streams. Research shows that even if the Ebbinghaus illusion is perceived, grip aperture is not affected by the illusion in neurotypical adults (e.g., Haffenden et al., 2001). Also, for the Ponzo illusion, it has been shown that grasping in neurotypical adults is not “fooled” by illusory displays (Ozana and Ganel, 2020). Studies on differences in perception and action relating to VIs have been used to demonstrate the dichotomy between dorsal and ventral streams. Research examining the functioning of ventral and dorsal visual streams in PD patients has revealed abnormalities in vision for action in a blind walking task coupled with intact performance on a line matching task (Giovannini et al., 2006). These findings suggest that impairments in visual perception in PD may be explained by abnormalities in dorsal stream processing, while the ventral stream remains unaffected, potentially preserving sensitivity to high-level VIs. In line with these findings, PD patients also experience deficits associated with higher level visual processing of motor actions including slower motor imagery (Poliakoff, 2013) and difficulties observing other people perform actions (Tremblay et al., 2007). These differences in processing visual action signal possible impairments in dorsal stream functioning.

This study is the first to test PD patients on their susceptibility to the Ebbinghaus, Ponzo, and Müller-Lyer illusions using the method of adjustment. PD patients and neurotypical age-matched controls completed a series of online illusion tasks in their own homes. On one hand, based on evidence of depth perception abnormalities in PD (e.g., Ou et al., 2018), we anticipated that PD patients may be less susceptible to these VIs than controls. However, we also believe the differences are likely to be stronger for VIs with most explicit depth, like the Ponzo illusion. However, on the other hand, we recognized that PD patients’ susceptibility to these VIs could be unaffected due to a lack of severe disruption to the ventral stream. Our findings will advance theoretical understanding of how PD impacts susceptibility to high-level VIs and ventral stream visual processing.

## Methods

### Participants

#### Power analysis

G*Power software (Faul et al., 2007) was used to perform an *a priori* power analysis to ascertain the necessary sample size required. Power (1-β) was specified as.80 and the significance level (α) was set to.05. The anticipated effect size was modeled on the results obtained by Grzeczkowski et al. (2018). Due to this, we anticipated a medium effect size of *d* = 0.46. For the frequentist parameters defined, a sample size of *N* = 56 is required to achieve a power of 0.80 at an alpha of.05. Hence, we aimed to recruit 56 participants.

#### Demographics

Participants included 27 PD patients (15 females, 12 males) and 28 neurotypical participants (17 females, 11 males). PD participants were recruited from the Department of Psychology database of PD patients at Lancaster University, while controls were recruited via convenience sampling (*n* = 18) and sign-ups to the Centre for Aging Research at Lancaster University (*n* = 10). All PD patients were medicated. Participants were predominantly white British (*n* = 47). Participants were largely well-educated, with the majority holding at least an undergraduate degree (*n* = 35). None of the participants reported having a cognitive impairment or any neurological illness. Nine participants reported having a psychiatric illness (anxiety: *n* = 5; 3 in the control group, and depression: *n* = 4; 3 in the control group). Eleven participants reported visual impairments for which they were receiving treatment, including glaucoma (*n* = 3; 1 in the control group), age-related macular degeneration (*n* = 2), double vision (*n* = 3), astigmatism (control group), keratoconus, and short-sightedness (control group; all *n* = 1). All participants confirmed that they had corrected-to-normal vision despite having these conditions, and the aforementioned difficulties did not affect their ability to perceive the VIs. Participants’ visual acuity was not assessed as previous research indicates that VIs susceptibility is not related to it (Cretenoud et al., 2021) as well as in PD visual acuity remains largely perseverated (Hunt et al., 1995).

No significant differences between PD patients and neurotypical controls were observed for age (*t* = 0.05, *p* = 0.96), years of formal education (*t* = 0.21, *p* = 0.835), scores for mild cognitive dysfunction (*t* = −0.706, *p* = 0.484), anxiety (*t* = 0.599, *p* = 0.07), and depression (*t* = 0.15, *p* = 0.882). These non-significant group differences indicate that the groups were closely matched (see Table 1 for more details). Full details of the PD patients’ cohort are presented in Table 2.

**Table 1:** Means and standard deviations for PD patients and neurotypical adults.

	Total	Age	Education	Depression	MOCA	Anxiety	Screen size
PD patients	27	63.3(7.64)	15.11(4.17)	4.67(2.73)	24.89(2.04)	5.78(3.94)	35.48(9.93)
Neurotypical adults	28	63.18(9.39)	14.89(3.52)	3.93(2.61)	24.54(2.04)	5.64(2.64)	36.93(4.3)

Higher values for depression and anxiety indicate more severe symptoms. Higher MOCA scores indicate better cognitive functioning. Screen size is reported in centimeters.

**Table 2:** Characteristics of PD patients.

Participant	Age	Gender	Years since the PD diagnosis	Years since PD onset	LEDD	Last dose	MOCA	HADS-A	HADS-D	Hoehn and Yahr stage
1	51	Female	3	5	555	204	22	4	1	1
2	62	Female	8	11	760	30	26	11	6	1
3	65	Male	5	5	660	148	25	1	2	0
4	63	Female	5	6	350	180	26	6	7	2
5	57	Female	2	6	375	85	25	15	6	2
6	56	Male	6	8	1,000	136	18	3	5	2
7	58	Male	2	3	973	120	26	6	7	1
8	74	Female	4	5	195	2	26	1	1	2
9	59	Male	5	15	220	0	23	2	3	1
10	70	Male	5	7	595	210	25	4	3	1
11	67	Male	5	10	960	720	25	1	5	1
12	67	Male	9	21	N.A.	204	26	3	0	2
13	70	Male	13	30	N.A.	90	26	2	2	2
14	71	Female	3	6	400	230	26	2	1	0
15	59	Male	4	7	590	25	26	4	3	2
16	67	Male	6	10	840	60	27	12	5	1
17	63	Male	4	5	475	240	25	7	6	1
18	59	Female	6	7	362	420	25	14	5	0
19	75	Female	7	7	1,680	127	25	5	7	2
20	51	Female	3	5	555	150	24	3	1	1
21	70	Female	11	2	578	0	27	6	3	2
22	57	Female	2	5	300	150	24	8	5	2
23	67	Female	5	6	500	120	26	6	8	1
24	51	Female	1	4	800	210	20	11	8	2
25	59	Female	5	16	355	1,440	26	5	9	1
26	81	Female	7	7	640	255	26	8	9	2
27	60	Male	4	6	715	45	26	6	8	3

The time since the last dose is in minutes. HADS-A and HADS-D correspond to anxiety and depression, respectively (described in further detail below). LEDD corresponds to L-dopa equivalent daily dose, which is among the most common medication for PD (Julien et al., 2021). Hoehn and Yahr’s scale refers to the severity of symptoms in PD, ranging from 0 (least severe) to 5 (most severe) (Goetz et al., 2008).

### Materials

All study stimuli were developed using Unity 3D© Gaming Engine and were visually displayed to participants using the “screen share” function in Microsoft Teams. The stimuli were modeled on existing work in the field (e.g., Chouinard et al., 2013; Sperandio et al., 2023). These studies were conducted virtually as a precaution to protect both participants and experimenters from COVID-19. Though it may be seen as a potential confound, previous research indicates that online testing yields reliable measurements, however, the effect sizes tend to be smaller (e.g., Chuey et al., 2021; Pallen et al., 2022). As participants viewed the stimuli through screen share on their personal devices, screen size ranged between 23 and 61 inches. An independent samples *t*-test indicated that screen sizes of PD patients (*M* = 35.48, *SD* = 10.56) and neurotypical controls (*M* = 36.93, *SD* = 4.30) did not significantly differ, *t*(53) = −0.706, *p* = 0.484. Also, no significant correlations were observed between illusion strength and screen size.

Three visual illusions were used: the Ebbinghaus illusion, the Ponzo illusion, and the Müller-Lyer illusion. Participants were required to adjust the size of a line or circle (depending on the illusion) until they perceived it as equivalent in size to the reference stimuli. The size was adjusted using the right and left arrow keys, and trials were progressed using the *ENTER* key. The experimental software obtained a measure of reaction time (ms). RT data was only used to detect skipped trials (responses faster than approximately 5 s, which were accompanied by large Z-score values, at least 2 standard deviations (SDs) away from the mean). Average RTs significantly differed between illusions [*F*(1.63, 88.05) = 5.37, *p* = 0.006] but not between participant groups [*F*(1, 54) = 1.12, *p* = 0.294]. *Post hoc* comparisons with Holm correction showed differences between RTs for the Ebbinghaus (*M* = 21.24, *SD* = 6.11) and the Müller-Lyer (*M* = 23.87, *SD* = 9.10) illusions, *t* = −2.75, *p* = 0.014, as well as between the Müller-Lyer (*M* = 23.87, *SD* = 9.10) and Ponzo illusions (*M* = 21.08, *SD* = 6.01), *t* = 1.92, *p* = 0.013. No difference was detected between RTs for the Ebbinghaus and Ponzo illusions; *p* = 0.867. Furthermore, we conducted correlations between the illusion’s strength and RTs for both groups individually, and the whole sample, to access if prolonged exposure affected VIs susceptibility (Bressan and Kramer, 2021). None of the correlations approached significance.

#### The Ebbinghaus illusion

**Figure 1:**
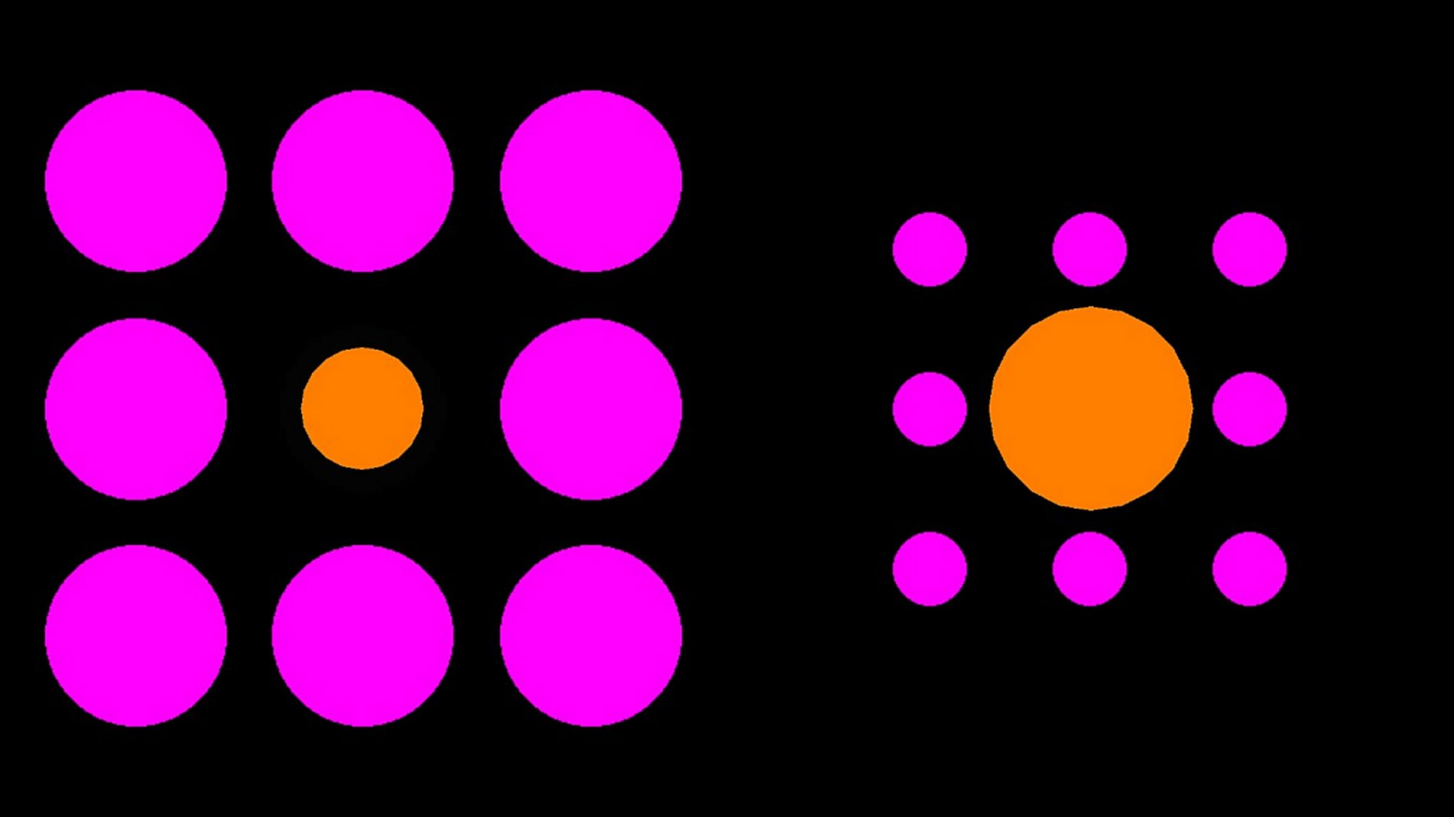
Example Ebbinghaus illusion trial. Participants were required to manipulate the size of the right orange circle to match the size of the left orange circle (or vice versa).

The two orange center circles were surrounded either by eight pink large inducers (125 pixels in diameter, positioned 35 and 90 pixels away from the central circle) or eight pink small inducers (50 pixels in diameter, positioned 32 and 80 pixels away from the central circle) presented on a black background (see Figure 1). The orange center circle was 100 pixels in diameter (an example display is illustrated in Figure 1). There were 16 trials in total. The starting size of the adjustable center circle was 50 pixels in 8 trials and 150 pixels in 8 trials. The side of appearance (left or right) and inducer size (large or small) for the adjustable circle varied between trials, with four trials for each size and side combination. The order of presentation was randomized.

#### The Ponzo illusion

**Figure 2:**
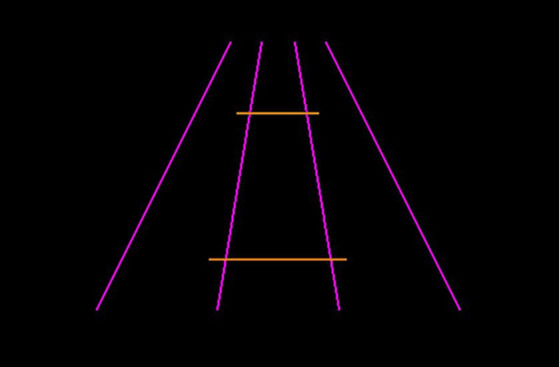
Example Ponzo illusion trial. Participants would be required to manipulate the length of the bottom orange line to match the length of the top orange line (or vice versa).

Four pink converging lines were used as inducers (two at 420 pixels in length at a 64-degree angle, and two at 380 pixels in length at a 10-degree angle). The adjustable and reference horizontal lines were orange and 135 pixels apart. The reference line for both methods of measurement was held constant at 100 pixels. An example display can be found in Figure 2. There were 8 trials in total; in 4 trials the adjustable line started at 50 pixels, and in 4 trials the adjustable line started at 150 pixels. In half of the trials, the adjustable line appeared above the horizontal midline and half below. The order of presentation was randomized.

#### The Müller-Lyer illusion

**Figure 3:**
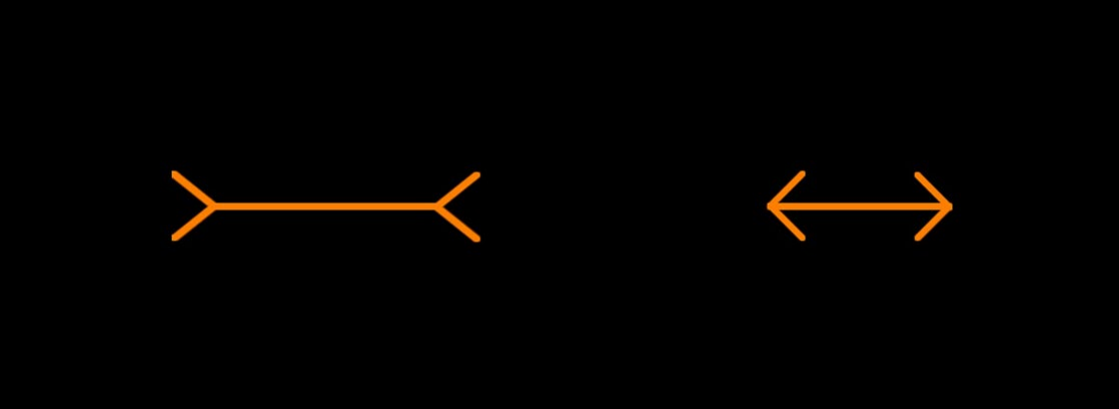
Example Müller-Lyer illusion trial. Participants would be required to manipulate the left orange line (between the arrowheads) to match the length of the right orange line (between the arrowheads), or vice versa.

Two orange lines with inwards or outwards facing arrows (40 pixels in length) at a 45-degree angle were presented. The reference line for both methods of measurement was held constant at 150 pixels. An example display can be found in Figure 3. There were 16 trials in total with four trials for each side of the presentation (left or right) and arrow type (inwards or outwards facing) combination. The starting size of the adjustable line was 75 pixels in 8 trials and 225 pixels in 8 trials. The order of presentation was randomized.

#### Questionnaires and screening tools

Questionnaires and screening tools were administered to participants via an online interview. These included the Hospital Anxiety and Depression Scale (HADS) (Snaith, 2003), the Montreal Cognitive Assessment (MOCA) (Nasreddine et al., 2005), and the Movement Disorder Society—Unified Parkinson Disease Rating Scale (MDS-UPDRS) (Goetz et al., 2007). These measures were included to test whether potential differences in susceptibility to VIs were influenced by participants’ cognitive abilities and/or mood.

HADS consists of 14 statements that measure traits of depression (7 items) and anxiety (7 items). Each statement has four corresponding answers which the interviewee can choose between. For example, for the statement “I feel tense or wound up” (an anxiety item), the response options are: “most of the time” (3 points), “a lot of the time” (2 points), “from time to time, occasionally” (1 point), and “not at all” (0 points). Higher scores indicate more severe symptomology. During the interview, the participant was instructed to think about their feelings over the past week. The statements were read out loud, followed by the answers, and then the participant chose one of them. If they were unsure, the interviewee was asked to make their best guess. For half of the questions the response options were read in order from negative to positive, and for the other half the response options were read in order from positive to negative.

The MOCA includes 13 tasks measuring a variety of cognitive functions, including visuospatial/executive functions, naming, memory, attention, language, abstraction, delayed recall, and orientation. As the study was conducted online, small changes were implemented. The first part of the visuospatial/executive task (connecting numbered dots) was omitted as the participant was unable to respond due to online administration. Also, in the orientation task, participants were not asked about their present location as the researchers were unable to validate their responses. The participant could therefore score up to 27 points (30 points originally).

The MDS-UPDRS consists of four subscales measuring: I—non-motor aspects of experiences of daily living (1.1–1.6) (questions 1.7–1.13 were excluded as they were unrelated to our study’s objective); II—motor aspects of experiences of daily living (2.1–2.13); III—motor examinations (3.1–3.8, 3.15–3.18) (questions 3.9–3.14 were dropped as the study’s online nature prevented the researchers from correctly assessing the participant’s performance); IV—motor complications (4.1–4.6). Parts I, II, and IV included questions asking participants to rate their difficulty engaging with a variety of daily tasks (e.g., getting dressed and getting out of a deep chair) from normal to severe on a five-point scale. Part III involved a motor examination of the participants, who performed tasks as they were described by the researcher (e.g., holding their hands still in front of them). The researcher then scored the performed action according to the MDS-UPDRS guidelines.

### Procedure

**Figure 4:**
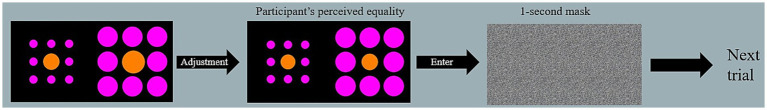
Example trial. During adjustment, the participant used the arrows on their keyboard to match the larger of the two orange, inner circles with the other, target circle. Once they perceived the circles as equal in size, they pressed enter to proceed to the next trial.

All participants were tested online via Microsoft Teams. Before taking part in the online session, participants were required to complete a survey requesting basic demographic information (e.g., age and gender), history of PD and diagnosis, and current medication intake. Then, all participants were screened for mild cognitive impairment (MOCA) and mood disorders (HADS). Individuals with PD symptoms were also assessed using the MDS-UPDRS. Participants were then given control over the researcher’s laptop using the Teams share function [which was not possible in some cases (<5), participants were asked to provide oral instructions to the researcher, however, our RT correlations with VIs susceptibility failed to reach significance, hence the different modes of entering data were not deemed problematic]. Once control was given, participants were presented with the experimental stimuli and asked to manipulate the size of a line (Müller-Lyer or Ponzo display) or center circle (Ebbinghaus display; either to increase or decrease) using the right and left (left to decrease, right to increase) arrow keys (see Figure 4). Once the participant believed that their stimulus matched the size of the reference non-adjusted line or circle, they were prompted to press *Enter*. If the participant was unable to take control, they were asked to orally instruct the researcher to either increase or decrease the sizes until they were happy with it. Participants were prompted to be as accurate as possible in their judgments and instructed to make their judgments as quickly as possible. In both scenarios, the researcher looked away from the screen to prevent the participant from feeling pressured to respond quickly or to prevent any gaze cues. The order of illusion blocks and trials within blocks were randomized. Once the experiment finished, participants were fully debriefed and encouraged to ask questions. The study took between 45 and 60 min to complete.

### Analysis plan

The data were screened to assess for normality of distribution. The magnitude of the illusion was calculated as the difference between the actual size of the target and the participant’s response. A 2 (Group: PD patients, neurotypical controls) × 3 (Illusion: Ebbinghaus, Ponzo, and Müller-Lyer) repeated measures ANOVA was conducted. Correlations between VIs, demographic data, and Parkinsonian symptoms were computed using both frequentist and Bayesian analyses. Multiple comparisons were analyzed with Holm correction (e.g., Grzeczkowski et al., 2018). Screening analyses were performed using IBM SPSS Statistics (IBM Corp, 2023; Version 27) and all the remaining analyses were performed in JASP Team (2023).

## Results

### Normality of the data set

Each participant’s data were screened for outliers (40 responses per participant) located at least two SDs away from the response mean (unusually low or high values reported), and compared against the population’s mean for each particular illusion. Outliers were screened for PD patients and neurotypical adults separately. To ensure consistency across responses, all individual outliers were replaced with a second value for the same trial type.

Several outliers were identified across the data. For the Ebbinghaus illusion, there were 27 outliers (3.01%) out of 896 trials, including 18 in the PD group (16 belonged to one participant, meaning every single trial of that participant was outside −/+ 2 SDs away from the mean, resulting in the exclusion of this participant) and 9 in the neurotypical group. For the Ponzo illusion, there were 20 outliers (4.46%) out of 448 trials, including 14 in the PD group and 6 in the neurotypical group. For the Müller-Lyer illusion, there were 31 outliers (3.45%) out of 896 trials, including 18 in the PD group and 13 in the neurotypical group. The majority of outliers were due to the participant pressing the *enter* key too forcefully, which resulted in skipping a trial (this was identified by unusually quick reaction times of less than 3 s). These scores were replaced with the participant’s second score in the same condition.

### Group differences between PD patients and neurotypical controls

**Figure 5:**
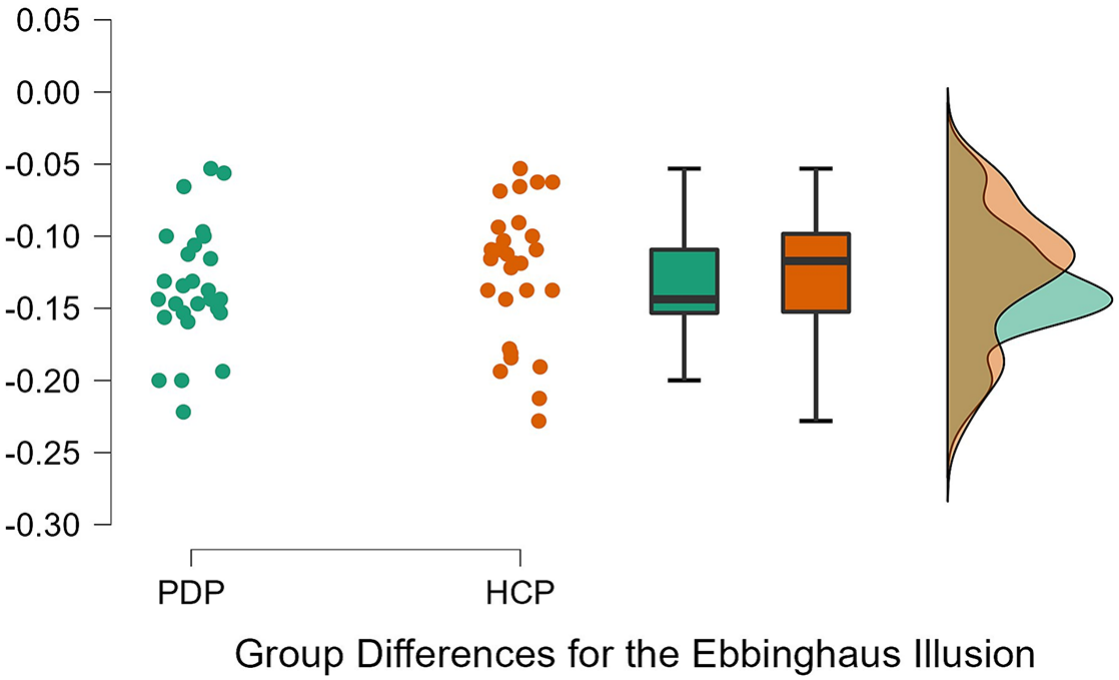
Individual data points for the Ebbinghaus illusion for PD patients (PDP) and healthy control participants (HCP). Both groups show overlapping similarities in their susceptibility to the Ebbinghaus illusion.

**Figure 6:**
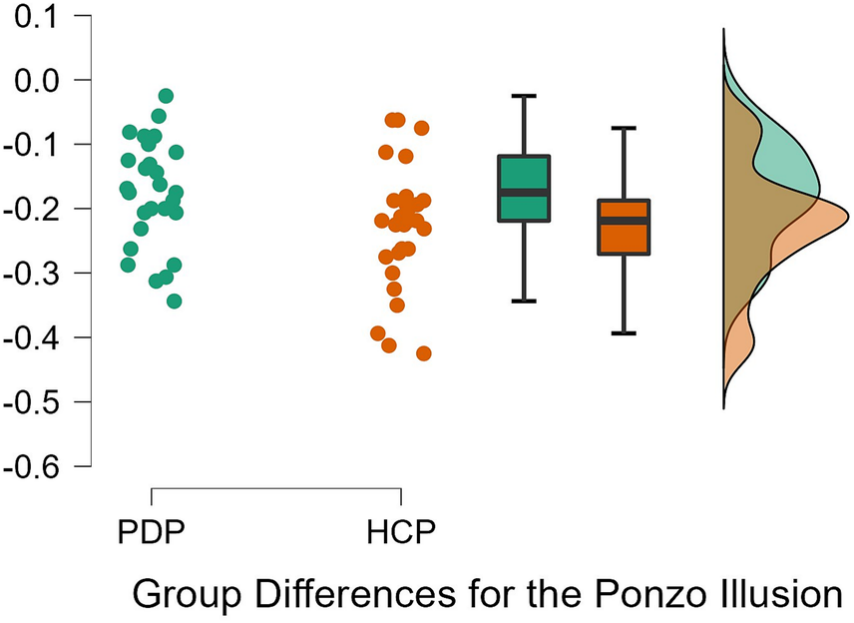
Individual data points for the Ponzo illusion for PD patients (PDP) and healthy control participants (HCP). Both groups show overlapping similarities in their susceptibility to the Ponzo illusion.

**Figure 7:**
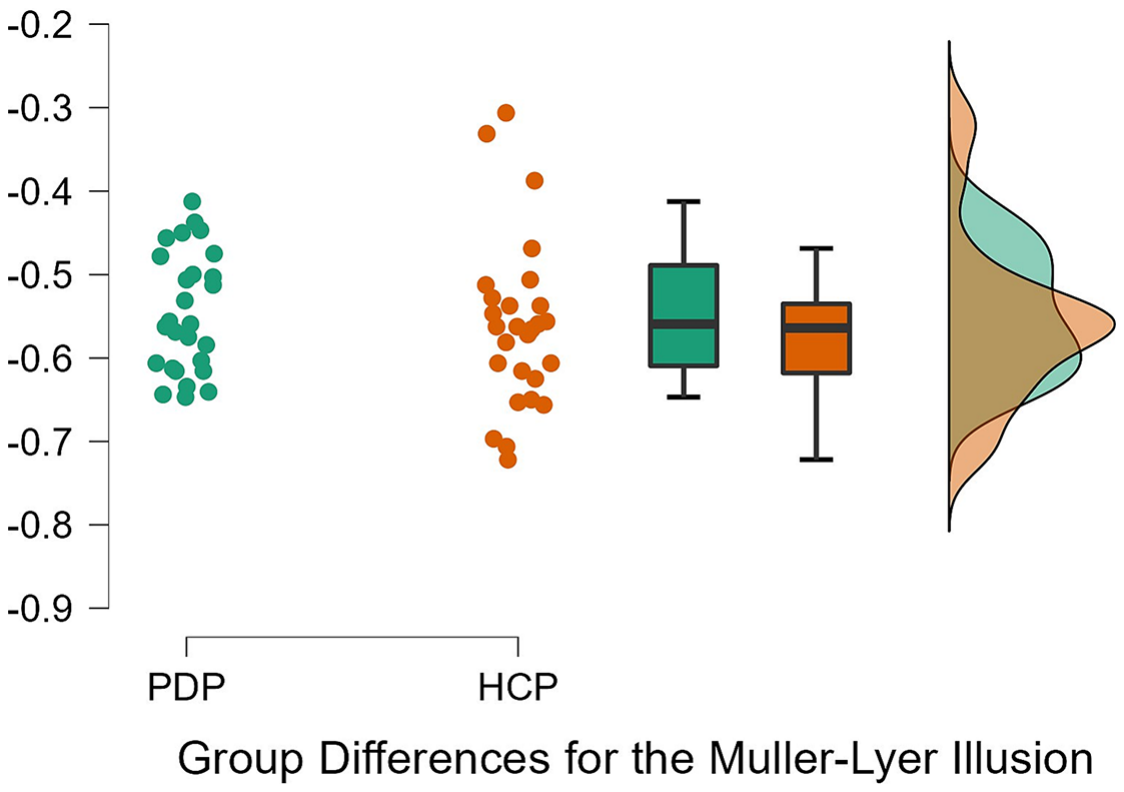
Individual data points for the Müller-Lyer illusion for PD patients (PDP) and healthy control participants (HCP). Both groups show overlapping similarities in their susceptibility to the Müller-Lyer illusion.

To examine differences between PD patients and neurotypical participants on their susceptibility to the Ebbinghaus, Ponzo, and Müller-Lyer illusions, a 2 × 3 repeated measures ANOVA was conducted. Both Levene’s test for equality of variance for all three illusions and Mauchly’s W test of sphericity indicated that the assumptions for a two-way ANOVA were met; *p* = 0.349, *p* = 0.777, *p* = 0.663, and *p* = 0.057, respectively. The results revealed a significant effect of the illusion, *F*(2, 108) = 628.63, *p* < 0.001, *η*^2^ = 0.87. The difference between PD patients and neurotypical approached significance, *F*(1, 54) = 3.79, *p* = 0.057, *η*^2^ = 0.003, as did the Population x Illusion interaction *F*(2, 54) = 3.07, *p* = 0.050, *η*^2^ = 0.004. Given our *a priori* predictions, we proceeded to conduct post-hoc comparisons though note that these should be treated with caution as the interaction was only marginally significant. Post-hoc comparisons using Holm correction (after Grzeczkowski et al., 2017) showed that PD patients were significantly less susceptible (*M* = −0.18, *SD* = 0.08) than controls (*M* = −0.23, *SD* = 0.09) to the Ponzo illusion; *t*(54) = 2.19, *p* = 0.033, *d* = 0.59. No significant differences were observed for the Ebbinghaus (PD; *M* = −0.14, *SD* = 0.04 and controls; *M* = −0.13, *SD* = 0.05) and Müller-Lyer illusions (PD; *M* = −0.54, *SD* = 0.07 and controls; *M* = −0.57, *SD* = 0.08) (Figures 5–7).

Similar results were observed by conducting a Bayesian 2 × 3 repeated measures ANOVA. Based on Jeffreys (1998) rule of thumb for interpreting Bayesian results (1–3, 3–10, and 10+, are considered weak, moderate, and strong effects, respectively), we observed weak evidence for an effect of VIs (BF = 0.89), very weak evidence for an effect of group (BF < 0.001), and weak evidence for an interaction (BF = 0.681). Bayesian *t*-tests yielded similar results for differences between the groups on each VIs. Weak evidence was observed for group differences on the Ebbinghaus, Ponzo, and Muller Lyer illusions; *B* = 0.399, *B* = 1.911, and *B* = 0.67, respectively. Evidence from these Bayesian analyses indicates a lack of differences between PD patients and neurotypical controls on the three tested illusions.

### Correlations

Several correlations were performed to assess whether severity of PD symptoms was associated with differences in susceptibility to VIs. The variables of interest included susceptibility scores for each illusion, time since the last medication dose, years since PD diagnosis, years since starting medication, years since symptom onset, LEDD score, and the total MDS-UPDRS score. As some variables were not normally distributed, Spearman’s correlations and their Bayes equivalent were conducted. No frequentist or Bayesian correlations approached significance, indicating that susceptibility to VIs was not correlated with patients’ PD characteristics.

## Discussion

This study investigated whether PD patients—a population characterized by basic and complex visual disturbances (e.g., Maschke et al., 2006)—and neurotypical adults differ in their susceptibility to the Ebbinghaus, Ponzo, and Müller-Lyer visual illusions. We formulated two competing hypotheses: (a) PD patients may be less susceptible to VIs than neurotypical adults due to abnormalities in the basal ganglia and dopamine deficits affecting their visual processing, or (b) sensitivity to VIs may not be impacted by PD due to their visual deficits specifically affecting dorsal stream processing of actions. Our analyses did not identify robust differences between the two populations’ responses for any illusion. These results suggest that dopamine deficiency and basal ganglia pathophysiology may not be directly related to VI susceptibility and that these may affect different aspects of visual perception (Maschke et al., 2006). Furthermore, our data imply that the ventral stream’s processing of vision for perception in PD is largely free from pathology when viewing VIs.

Previous research has shown that depth perception deteriorates in older adults (Salonen and Kivela, 2012) and that the inability to perceive depth correctly increases their risk of falls (Cummings et al., 1995; Ivers et al., 2000; Lord and Dayhew, 2001). There is also an extensive body of evidence documenting abnormal depth perception in PD (e.g., Ou et al., 2018), including in illusory contexts (Maschke et al., 2006). Our analysis, however, showed only marginal evidence for abnormal depth perception. PD patients appeared to have reduced susceptibility to the Ponzo illusion. The Ponzo illusion is considered a classic example of a depth illusion (Gregory, 1963), and creates the most apparent experience of depth among the tested illusions. These findings suggest that dopamine deficiency and/or pathophysiology of the basal ganglia may, marginally, affect depth perception as shown by the illusory depth in the Ponzo illusion, adding to already existing evidence concerning such deficits (e.g., Maschke et al., 2006). It is, however, important to note that the depth here is only illusory (induced), and arguably less apparent compared to the Ames Window illusion (such as in Maschke et al., 2006), and it is not real, 3D depth. PD patients might still have difficulties in perceiving depth in everyday situations (e.g., Cummings et al., 1995). Potentially, only a slight indication of reduced susceptibility was observed because PD participants in this study were mostly in the early- and mid-stages of PD. Therefore, it might still be possible that susceptibility to VIs starts deteriorating as PD develops, as other aspects of vision like color and contrast discrimination abilities get progressively worse (Diederich et al., 2002).

Reduced ability to interpret and process depth cues may result in abnormal susceptibility to the Ponzo illusion. Thus, an incorrect perception of an object’s position in the world (whether it appears as closer/further away than it is), could contribute to the increased risks of falls in the elderly. In line with this assumption, many PD patients are shown to exhibit difficulties in perceiving depth, experiencing both teleopsia (objects appear to be further away than they are) and pelopsia (objects appear to be closer than they are; Sasaki et al., 2021). Furthermore, it is unlikely that these differences observed between PD patients and controls arise due to the abnormal role of top-down influences in susceptibility to the Ponzo illusion, as such a deficit should also be observed for the Ebbinghaus illusion, which is considered a context sensitivity illusion (Káldy and Kovács, 2003).

The Ebbinghaus illusion arises due to the perceptual system’s top-down integration of display elements (Káldy and Kovács, 2003). Our data show that susceptibility to the Ebbinghaus illusion is not significantly different in PD, indicating typical abilities to integrate context in this population. This finding aligns with previous research reporting intact top-down influences on PD patients’ responses in visual priming tasks (Straughan et al., 2015) and visual search tasks (Horowitz et al., 2006). By contrast, Mannan et al. (2008) found that PD patients were impaired in visual search tasks involving highly salient targets, indicating difficulties with bottom-up processing. The illusions tested in this study belong to a category of high-level VIs that rely on complex cognitive processing and top-down mechanisms, whereas low-level VIs (e.g., the Brightness illusion) are mediated at the level of the retina and bottom-up perception (King et al., 2016). While PD may not impact top-down processing involved in experiencing complex VIs, deficiency of retinal dopamine may result in abnormal susceptibility to low-level VIs. As deficiency in retinal dopamine results in a diminished ability to differentiate contrast (as in color, e.g., Price et al., 1992; Pieri et al., 2000), PD patients could have higher thresholds in matching color in Brightness or Adelson’s Checkerboard illusions. Therefore, we recommend that future research investigates whether susceptibility to low-level VIs is affected by PD.

Our findings suggest that the pathophysiology of the basal ganglia and dopamine deficits may not affect PD patients’ sensitivity to the Müller-Lyer illusion. Therefore, illusions such as the Ebbinghaus and Müller-Lyer may be subserved by neural mechanisms that are largely free from pathophysiology in PD, such as those located in the visual cortex (Cheng et al., 2011; King et al., 2016). The Müller-Lyer illusion is considered to rely on depth cues (Gregory, 2015), just like the Ponzo illusion, which is considered a classic example of a depth illusion (Gregory, 1963). Therefore, the inability to perceive depth cannot be a major factor driving the illusion, at least in the version used here. In line, with Doherty et al. (2010) claims that subtle depth cues are likely to play a part in susceptibility to the Ebbinghaus illusion, the depth cues in the Müller-Lyer illusion are also subtle, hence no differences in susceptibility to those two illusions might have been observed. Thus, the pathophysiology of the basal ganglia and/or dopamine deficits might only be related to more explicit perceptions of depth, and are not directly linked with susceptibility to the Müller-Lyer illusion.

Overall, our observed results support the alternative hypothesis that susceptibility to VIs is largely unaffected in PD patients due to their visual perception difficulties originating from abnormalities in dorsal stream functioning, rather than ventral stream functioning. PD patients showed similar susceptibility to the Ebbinghaus and Müller-Lyer illusions and only marginal evidence for reduced susceptibility to the Ponzo illusion was observed. From this, we conclude that perception of depth is more crucial for executing motor actions than the integration of context. This is, in line with findings by Giovannini et al. (2006) who observed that PD patients display abnormalities in their vision for action in a blind walking task, but not a line-matching task. Arguably, the line-matching task does not rely on depth integration, therefore PD patients performed similarly to controls.

Extending this line of research to grasping behavior, which is guided by the dorsal stream, would potentially provide valuable insight into differences between the dorsal and ventral streams in PD. Previous findings on the dichotomy between the two streams have largely focused on whether individual illusory effects are larger on the ventral stream than the dorsal stream. Here, testing PD patients would allow for a different perspective; one would still assume that the perceptual stream is affected by the illusion in both PD patients and healthy controls, but the action stream is affected by the illusion only in PD patients.

This study has several limitations. Firstly, we did not directly assess our participants’ dopamine levels or pathophysiology of the basal ganglia. In line with other studies in the field (e.g., Maschke et al., 2006), our target population was selected based on robust pre-existing knowledge that PD is characterized by dopamine loss and basal ganglia pathophysiology which are known to adversely affect visual perception. Therefore, our conclusions that dopamine loss and the pathophysiology of the basal ganglia do not influence susceptibility to high-level VIs should be interpreted with caution. Furthermore, the online administration of the study resulted in several potential shortcomings. First, varying Internet speed could cause a lag in the delivery of the experiment, impacting the smoothness of the increase/decrease of the targets which the experimenter could not control for. Secondly, although participants were frequently reminded to rely on their visual perception alone, the experimenter could not verify whether the participants truly did so. Finally, our study did not check for the presence of everyday VIs (that are similar to geometrical VIs, but they occur during everyday activities of the patients), that recently gained interest in medical research on PD (Nishio et al., 2018; Sasaki et al., 2021).

In conclusion, our findings suggest that PD patients and neurotypical controls do not differ in their susceptibility to the Ebbinghaus, Ponzo, and Müller-Lyer illusions. The lack of differences was especially evident in the Ebbinghaus and Müller-Lyer illusions that more strongly rely on context sensitivity rather than depth perception. Only a marginal indication of abnormalities in depth perception was indicated by reduced susceptibility to the Ponzo illusion, which compared to the other VIs is a classical illusion of depth. Collectively, our data suggest that context integration, a key component of VIs susceptibility, remains unaffected in the early to mid-stage of PD. Furthermore, our findings suggest that visual deficits in PD are more likely to be related to the dorsal visual stream. This study makes a novel contribution to a growing literature exploring visual deficits in PD and advances the understanding of how visual perception may be affected by dopamine deficiency and abnormalities in the basal ganglia.

## Data availability statement

The raw data supporting the conclusions of this article will be made available by the authors, without undue reservation.

## Ethics statement

The studies involving humans were approved by the Lancaster University Faculty of Science and Technology Ethics Committee. The studies were conducted in accordance with the local legislation and institutional requirements. The participants provided their written informed consent to participate in this study.

## Author contributions

RW: Conceptualization, Data curation, Formal analysis, Investigation, Methodology, Project administration, Validation, Writing – original draft, Writing – review & editing. CH: Supervision, Writing – review & editing. MR: Conceptualization, Investigation, Project administration, Resources, Writing – review & editing. SL: Methodology, Software, Supervision, Validation, Writing – review & editing. TC: Conceptualization, Supervision, Writing – review & editing.
